# The autoantibody-mediated encephalitides: from clinical observations to molecular pathogenesis

**DOI:** 10.1007/s00415-019-09590-9

**Published:** 2019-10-26

**Authors:** Sudarshini Ramanathan, Adam Al-Diwani, Patrick Waters, Sarosh R. Irani

**Affiliations:** 1 Oxford Autoimmune Neurology Group, Nuffield Department of Clinical Neuroscience, John Radcliffe Hospital, Oxford, UK; 2 University of Oxford, Oxford, UK; 3 Sydney Medical School, University of Sydney, Sydney, Australia; 4 Kids Neuroscience Centre, Children’s Hospital at Westmead, Sydney, Australia; 5 Department of Psychiatry, Warneford Hospital, University of Oxford, Oxford, UK; 6 Department of Neurology, Oxford University Hospitals NHS Foundation Trust, Oxford, UK

**Keywords:** Autoimmune encephalitis, NMDAR encephalitis, LGI1 encephalitis, Neuroimmunology, Autoantibodies, Seizures

## Abstract

The autoimmune encephalitis (AE) syndromes have been characterised by the detection of autoantibodies in serum and/or cerebrospinal fluid which target the extracellular domains of specific neuroglial antigens. The clinical syndromes have phenotypes which are often highly characteristic of their associated antigen-specific autoantibody. For example, the constellation of psychiatric features and the multi-faceted movement disorder observed in patients with NMDAR antibodies are highly distinctive, as are the faciobrachial dystonic seizures observed in close association with LGI1 antibodies. These typically tight correlations may be conferred by the presence of autoantibodies which can directly access and modulate their antigens in vivo. AE remains an under-recognised clinical syndrome but one where early and accurate detection is critical as prompt initiation of immunotherapy is closely associated with improved outcomes. In this review of a rapidly emerging field, we outline molecular observations with translational value. We focus on contemporary methodologies of autoantibody detection, the evolution and distinctive nature of the clinical phenotypes, generalisable therapeutic paradigms, and finally discuss the likely mechanisms of autoimmunity in these patients which may inform future precision therapies.

## Introduction

For over 5 decades, the clinical syndrome characterised by the subacute onset of amnesia, agitation, confusion, hallucinations, seizures and sleep disturbance, often accompanied by medial temporal lobe signal changes on imaging, has been referred to as limbic encephalitis (LE) [1–3]. Some patients with LE have defined autoantibodies which are not thought to be directly pathogenic, and target intracellular onconeural antigens including nuclear or cytoplasmic proteins (such as Hu, Ma, Ri, and Yo) [2]. These patients often have underlying malignancies and these are likely dominantly T cell-mediated conditions [4–6].

By contrast, in recent years, a group of autoimmune encephalitis (AE) syndromes have been characterised by the detection of autoantibodies in serum and/or cerebrospinal fluid (CSF) which target the extracellular domains of specific neuroglial cell-surface antigens [2, 3, 7–10]. These autoantibodies can access their target antigens in vivo, and it is now widely accepted that their disruption of the target antigen results in the observed neurological sequelae [6, 7, 11–14]. Therefore, these antibodies have pathogenic potential and their early and accurate detection is critical for two main pragmatic clinical reasons. First, the incidence of autoantibody-mediated encephalitis is equivalent to that of infectious encephalitis [15]. In our clinical experience, it is likely to exceed the frequency of infectious causes, when the seronegative forms of AE are also taken into account. Second, while the clinical manifestations may range from mild to life threatening, the syndromes are typically responsive to immunotherapy [7], with early treatment being consistently identified as a factor in improved long-term outcomes [8, 16, 17]. Hence, this review will summarise features which should encourage early clinical recognition and outline current treatment strategies. In addition, we discuss autoantibody detection methods and describe contemporary insights into the neuroscience and cellular immunology which underlie these fascinating conditions.

## Relative merits of autoantibody detection methods

**Fig. 1:**
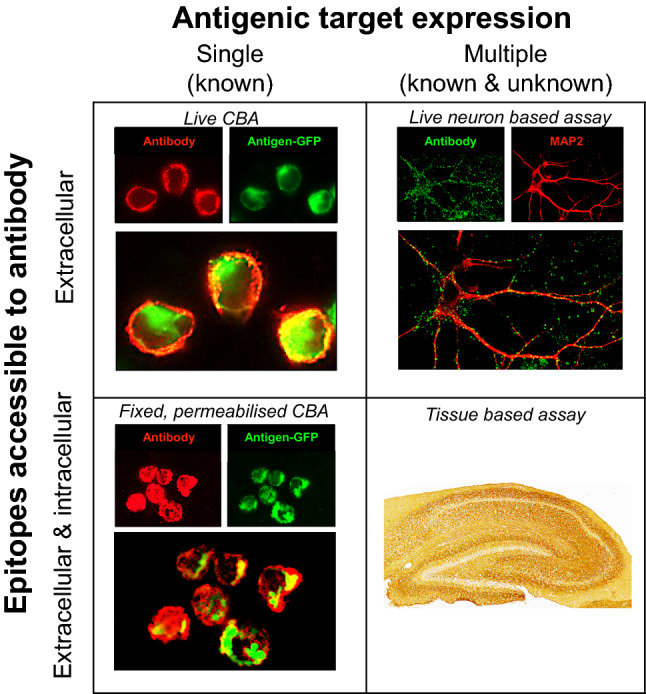
Neuronal surface antibody detection methods. Prevailing methods used in research and diagnostic practice expose neuronal antigens to the test sample but differ in the properties of the antigen(s). Cell-based assays (CBA) aim to largely expose a single known antigen, by cell transfection. Conversely, neuron-based assays and tissue-based assays expose multiple natively expressed antigens which include those known to be targets of pathogenic antibodies, in addition to as-yet unknown antigens. Additionally, the assays vary on whether the antigen is fixed prior to incubation with the sample, and whether the cell membrane is intact. Live CBAs and live neuron-based assays neither fix the surface antigen nor permeabilise the membrane prior to exposure to the patient’s sample. In contrast, in fixed permeabilised CBAs and tissue-based assays, target antigens are fixed and cell membrane integrity is lost. *CBA* cell-based assay

All autoantibodies which mediate AE bind conformational antigenic epitopes. The tertiary structure of the target protein is thought to create distinctive three-dimensional domains to which autoantibodies preferentially bind. Conversely, loss of this structure reduces the likelihood of binding and subsequent detection of potentially pathogenic autoantibodies. This principle has underpinned the development of assays used in discovery phase research as well as those optimised for routine diagnostic testing (Fig. 1).

HEK293 cells provide a robust system for expressing proteins in their native conformational state on a mammalian surface membrane. As a result, they are a key reagent in the successful mammalian expression of the conformationally active extracellular domains of the known target antigens. Indeed, they are commonly employed in cell-based assays (CBAs), and have roles both in research to test for candidate targets, and in diagnostic testing to specifically identify a clinically suspected autoantibody. The patient serum or CSF can be applied to the transfected HEK cells either when they are live and intact (‘live CBA’) or after fixation and permeabilization (‘fixed CBA’). Live CBAs avoid permeabilisation and fixation-induced disruption of autoantigens, so patient autoantibodies are only exposed to the most clinically relevant extracellular epitopes. In our experience, this testing approach contributes to higher sensitivity for detecting immunotherapy-responsive conditions [18–21]. However, the cells must be cyclically prepared and used within a narrow time window, limiting their availability to expert laboratories. By contrast, fixed CBAs expose potentially denatured and intracellular antigens, but can be easily disseminated to other labs and used after preparation for several months.

Live cultured rodent neurons or fixed brain slices provide substrates for ‘neuron-based assays’ or ‘tissue-based assays’, respectively. These preparations express the native epitopes from neurons, rather than those expressed by non-neuronal, albeit mammalian, cells such as HEK cells. Therefore, they may further improve the detection of autoantibodies with pathogenic potential. Theoretically, they can be used to screen for binding to any endogenous neuronal antigen, although fixed slices will of course detect antibodies which bind intracellular targets. Additionally, they may yield highly characteristic binding patterns and provide information about subcellular and whole brain localisation of autoantigens. These approaches depend on considerable technical expertise to optimise antigen availability, autoantibody detection, and image interpretation. Consequently, their main role remains in research laboratories for autoantibody discovery, or as an adjunct to confirm CBA-determined autoantibody specificities in more native tissue [3, 21].

## Use of serum versus CSF

The relative value of serum versus CSF for testing provides an interesting set of challenges. Serum has both a high level of IgG (around 10 mg/mL) and a huge diversity of antibody-binding specificities. Hence, serum testing is inherently challenging, compared to the approximately 500-fold lower total IgG in CSF. However, as nearly all potentially pathogenic antibodies have a higher concentration in serum compared to CSF, likely reflecting their initial peripheral generation, serum findings have biological and diagnostic importance. Particularly in serum, high-sensitivity assays permit the detection of target-specific conformational antibodies that are of low affinity and/or at a low level [22, 23]. However, the detection of these lower affinity/level antibodies may increase the likelihood of detecting autoantibodies which lack direct clinical relevance with respect to the canonical AE syndrome in question. Yet, these findings appear to be biochemically robust in defining the presence of antigen-specific reactivities [20, 22, 23]: they do not appear to be false-positive results. Hence, we prefer the term *clinically irrelevant* as they do not currently appear directly translatable to the level of individual patients. In contrast, CSF provides a ‘cleaner’ sample for autoantibody determination and in many cases of AE there is a relative overproduction of the antigen-specific autoantibodies in the intrathecal compartment (‘intrathecal synthesis’), as reflected in the high frequencies of antigen-specific B cells in CSF [2, 24–26]. For some AE autoantibodies, this confers CSF with low background, and it can often be tested with minimal dilution to boost sensitivity without concern about loss of specificity. However, some AE autoantibodies—such as those against LGI1—are preferentially detected in serum versus CSF in clear-cut clinical cases [27] (Michael, Ramberger, and Irani, unpublished observations). Therefore, overall, the literature and our experience suggest that paired serum–CSF testing in the context of a clear pre-test clinical probability forms the basis of a reliable diagnostic approach. This a priori clinical hypothesis remains the cornerstone of successful diagnostic testing and is, therefore, the focus of the next section.

## Clinical features of the neuronal surface autoantibody-mediated encephalitis syndromes

*N*-Methyl-d-aspartate receptor (NMDAR) and leucine-rich glioma-inactivated 1 (LGI1) antibodies define the most prevalently recognised AE syndromes, and will be the primary focus of this review (Table 1). In addition, over the last decade, numerous other CNS antigens have been identified as targets in less frequent, clinically varied forms of AE. These autoantibodies are directed against the γ-aminobutyric acid A and B receptors (GABA_A/B_), α-amino-3-hydroxy-5-methyl-4-isoxazolepropionic acid receptor (AMPAR), glycine receptor (GlyR), dipeptidyl-peptidase-like protein-6 (DPPX), dopamine 2 receptor (D2R), metabotropic glutamate receptor 5 (mGluR5), neurexin-3α, and IgLON5. The demographics, clinical presentations, paraclinical investigations, and the therapeutic responses of these individual syndromes are summarised in Table 2. Much of this clinical information is based on retrospective diagnoses and relatively small cohorts. Nevertheless, almost all studies still point towards a consistent paradigm of an autoantibody-medicated encephalitis occurring over an acute or subacute duration, often with associated impaired cognition, neuropsychiatric manifestations, seizures, movement disorders, and sleep dysfunction, with or without a tumour association; and a high likelihood of response to immunotherapy.

**Table 1:** NMDAR- and LGI1-antibody encephalitis: a comparison

	NMDAR antibody	LGI1 antibody
Protein antigen [26, 54, 60, 61, 90]	N-terminal domain on extracellular aspect of NR1 (GluN1) subunit of the NMDA receptor	LGI1 is secreted into the synapse and interacts with two synaptic proteins of the disintegrin and metalloproteinase domain (ADAM) family—presynaptic ADAM23 and postsynaptic ADAM22
Demographic [8, 16, 106]	Throughout life span, but most commonly younger women Modal onset: 18–23 years; range: infancy–89 years	Largely older adult men Modal onset: 64 years; range: 22–92 years
Tumour association	Well-defined association with ovarian teratoma in young adult patients; overall rate ~ 25%. Other varied tumours are rare	Most do not have an associated tumour. Up to 10% have thymoma. Other tumours are rare.
Prodrome [8, 60, 72, 76]	Flu-like (headache, malaise, mild fever, gastrointestinal symptoms)	FBDS and other seizure semiologies typically precede cognitive impairment
Time course	Usually abrupt or subacute onset Progressive: Prodrome → psychiatric → neurological → critical illness.	Subacute onset, typically If not treated during seizure only phase, often progresses to full encephalitis
Psychiatric [107]	Almost all adult patients, less dominant in childhood cases. Polymorphic syndrome involving multiple psychopathological domains, e.g. sleep, mood, psychosis, behavioural and catatonia	Behaviour/personality change common (~ 90%). Can include irritability, disinhibition, compulsivity, emotionality, and insomnia
Neurologic [46]	Seizures common (~ 70%) but rarely frequent Movement disorder in > 90%. Often multiple simultaneous phenomenologies with a dominant triad of dystonia, stereotypies and chorea. Orofacial dyskinesia are common Dysautonomia	Frequent seizures are very common (~ 90%), with FBDS in 50% Non-FBDS seizures semiologies diverse and are commonly focal; generalised tonic–clonic seizures less common and in latter stages Cognitive impairment
CSF	Cell count: lymphocytic pleocytosis and unpaired oligoclonal bands common. Routine CSF analysis normal in ~ 20%	Cell count: lymphocytic pleocytosis and unpaired oligoclonal bands possible but normal in ~ 75%
EEG [108]	Usually generalised > focal slowing (~ 80 to 90%); in severe cases consistent with extreme delta brush	Mild diffuse slowing, ictal and interictal abnormalities with non-FBDS focal seizure semiologies
Brain MRI [8, 27, 78]	Often normal (~ 70%) despite severe illness: ‘clinical-radiologic paradox’	Around 50% show medial temporal lobe swelling with T2/FLAIR hyperintensities. FBDS associate with basal ganglia changes
Other blood tests	Creatine kinase can be elevated	Serum hyponatraemia in around 50%; > 90% association with HLA- DRB1*07:01
Outcomes [8, 16]	Overall: mortality up to 15%, relapse rate up to 30%. With immunotherapy, > 75% of patients with mRS < 3	Overall: mortality < 5%, relapse rate up to 30%. Immunotherapy (particularly steroids) but not antiepileptic drugs prevent progression of FBDS to cognitive impairment. With immunotherapy, > 80% have mRS < 3

*ADAM* a disintegrin and metalloproteinase domain, *CSF* cerebrospinal fluid, *EEG* electroencephalogram, *FBDS* faciobrachial dystonic seizures, *FLAIR* fluid-attenuated inversion recovery, *LGI1* leucine-rich glioma-inactivated 1, *MRI* magnetic resonance imaging, *mRS* modified Rankin Scale, *NMDA N*-methyl-d-aspartate receptor

**Table 2:** Characteristics of autoimmune encephalitis associated with neuronal surface antibodies

Neuronal surface target	Antigenic target characteristics	Demographics (age/gender)	Clinical presentation	Investigations	Coexistent antibodies	Paraneoplastic association	Relapse rate and outcomes
CASPR2 [60, 62, 64, 82, 109]	Cell adhesion molecule which colocalises with Kv1.1 and Kv1.2 at the neural juxtaparanodes in both the CNS and PNS	Median age 60 s-70 s; M:F 9:1	Neuromyotonia, neuropathic pain (up to 40%), muscle cramps/fasciculations; LE; Morvan’s syndrome with neuropsychiatric changes, dysautonomia, sleep disturbance (insomnia, agrypnia excitata) and neuromyotonia	CSF normal in up to 70% Imaging normal in up to 70%. May have T2 hippocampal hyperintensities as with LGI1 antibodies Association with HLA- DRB1*11:01	Can be present concurrently with LGI1 and contactin-2 antibodies in Morvan’s syndrome	12-50%; mainly thymomas in Morvan’s syndrome; also, lung cancer, endometrial adenocarcinoma	>80% have favourable responses to immunotherapy – especially in absence of a tumour; 10% mortality rate; up to 30% relapse rate
GlyR [6, 92, 110–112]	Ionotropic receptor with five subunits (α1–4 and β); facilitates inhibitory neurotransmission in the brainstem and spinal cord; antibodies target the α subunit	Median age 40 s, range (3-70 s); roughly equivalent gender distribution in adults	Progressive encephalomyelitis with rigidity and myoclonus (PERM), over 10% of stiff person syndrome, epilepsy	CSF usually normal, ~ 50% may have inflammatory changes MRI largely normal, few patients may have signal change in temporal lobes, and patchy or longitudinal involvement of spinal cord	Coexistent GAD65 antibodies in some patients	Up to 10%; thymoma, lymphoma, metastatic breast cancer	Generally, respond well to immunotherapy, with GlyR antibody stiff person syndrome being more immunotherapy responsive than seronegative cases; may relapse in 15%; 10% mortality rate
GABA_A_R [93, 113, 114]	Ionotropic receptor which mediates fast inhibitory synaptic transmission; relevant antibodies target the α1, b3, and γ2 subunits	Median age in 40 s (range 2 months-88 years); M:F 1:1	Encephalitis with severe seizures (inclusive of status epilepticus, epilepsia partialis continua); confusion, disorientation, hallucinations and other psychiatric features, cognitive dysfunction, movement disorders; lower titres associated with stiff person syndrome, opsoclonus-myoclonus	Many have CSF lymphocytic pleocytosis ± oligoclonal bands and elevated protein; few may be normal Frequently have FLAIR and T2 abnormalities on MRI, usually multifocal or diffuse “fluffy” cortical and subcortical involvement	Coexistent antibodies to GAD65, GABA_B_R > LGI1, NMDAR, and thyroid peroxidase	< 20% with tumours including thymomas, non-Hodgkin lymphoma, SCLC, rectal cancer	Over 80% may respond to immunotherapy ± tumour removal; but full recovery in only 30%; up to 20% mortality rate (especially in context of status epilepticus); relapses in 10%
GABA_B_R [6, 24, 115–117]	Neuronal synaptic G protein-coupled receptor involved in inhibitory synaptic transmission	Median age 60 s, range 16-75; M:F 1.5:1	LE, with a prominent seizure phenotype (often temporal with secondary generalisation; status epilepticus), confusion, memory loss	CSF lymphocytic pleocytosis common. Frequently have unilateral or bilateral medial temporal lobe T2 hyperintensity on MRI, may be normal	Coexistent antibodies to GAD65, thyroid peroxidase, N-type voltage-gated calcium channels, Hu, CV2, and SOX1 in some patients	Tumour association ~ 50%; SCLC in up to half the patients	Some patients are immunotherapy responsive, poor outcomes largely attributed to underlying malignancy; up to 10% may relapse, mortality in up to 40%
AMPAR [6, 91, 117]	Ionotropic glutamate receptor made up of four subunits (GluR1-4); critical in synaptic plasticity and excitatory neurotransmission; antibodies directed against GluR1/2 subunits	median age mid 50-60 s; range young adults-90 s; M:F 1:2.5	LE, encephalopathy, confusion, seizures, cognitive impairment, amnesia, disordered sleep, movement disorders	May show CSF lymphocytic pleocytosis ± oligoclonal bands, may be normal Frequently have unilateral or bilateral medial temporal lobe hyperintensity on MRI, atrophy at follow up; may be normal		In 50%, SCLC, breast, thymic, and ovarian cancers	Most patients show a partial response to oncological management and immunotherapy responsiveness; relapses appear common
mGluR5 [6, 118, 119]	Involved in hippocampal synaptic depression	Median age late 20 s (range 6-75): M:F 1.5:1	LE, cognitive impairment, memory deficits, confusion, psychiatric symptoms; ‘Ophelia syndrome’ in context of Hodgkin lymphoma	May show CSF lymphocytic pleocytosis MRI may be normal in half		Hodgkin lymphoma	Generally responsive to treatment of Hodgkin lymphoma and immunotherapy
DPPX [120–122]	Extracellular subunit of the Kv4.2 potassium channel, influences potassium channel gating in cerebellum, hippocampus, and myenteric plexus	Median age 50 s-60 s (range 13-76); M:F 2.5:1	Prodrome of severe diarrhoea and weight loss. Subacute onset of cognitive impairment, agitation, confusion, hallucinations, seizures, sleep dysfunction; tremor, hyperekplexia, myoclonus; bulbar dysfunction and autonomic dysfunction	CSF lymphocytic pleocytosis ± elevated protein and oligoclonal bands, but may be normal Imaging mostly non-specific changes		B cell neoplasms in < 10% (such as gastrointestinal follicular lymphoma, chronic lymphocytic leukaemia)	May have multiple relapses in close to 25%; 60% can respond partially or significantly to (often intensive) immunotherapy, mortality 17%
D2R [123, 124]	Postsynaptic receptor with striatal expression, important in dopaminergic neurotransmission and motor control; antibodies bind to amino acids 20-29 and 23-37 of N-terminus	Median age 6 years (range 1-15); M:F 1:1; high proportion of patients of non-Caucasian ethnicity	Parkinsonism, dystonia, chorea, hypersomnolence, neuropsychiatric features (obsessive compulsive disorder, psychosis, emotional lability), ‘basal ganglia encephalitis’	CSF may show lymphocytic pleocytosis and/or oligoclonal bands 50% have MRI changes including basal ganglia swelling, hyperintensity or enhancement acutely; and atrophy and gliosis on follow up		No associated cancer	Immunotherapy responsive, 25% may relapse
IgLON5 [104, 125–127]	Member of the immunoglobulin superfamily of cell adhesion molecules in neurons	Median age 60 s (range 13-80 s); M:F 1:1	Progressive dyssomnia, movement disorders and behaviour, gait abnormalities, bulbar and respiratory dysfunction, and cognitive impairment; disease onset often more insidious compared to other autoimmune encephalitis syndromes	CSF may be non-contributory or may show lymphocytosis in third, elevated protein in half; oligoclonal bands rare MRI changes may be non-specific Histopathologically characterised by neuronal accumulation of hyperphosphorylated tau involving hypothalamus and brainstem, and associated neuronal loss, gliosis, and absence of inflammatory infiltrate Strong HLA Class II association		Unknown	Severe and progressive, with early reports stating > 70% mortality and minimal response to immunotherapy; later series identify broader phenotype and show immunotherapy may result in improvement and stabilisation
Neurexin-3α [128]	Presynaptic cell adhesion molecule which plays a role in synapse formation and maturation	Median age 44, range 23-57; M:F 1:4	Prodromal fever, headache, gastrointestinal symptoms; subsequent encephalopathy with agitation, seizures, orofacial dykinesias, and central hypoventilation (marked overlap with NDMAR encephalitis); may have a rapid course	CSF lymphocytic pleocytosis in most Imaging may be normal or may show FLAIR/T2 temporal lobe abnormalities		Unknown	Severe syndrome but only one case series to date
GluRD2 [129]	Cerebellar expressed ionotropic receptor with a role in synaptic organisation	Paediatric onset 12-36 months; M:F 1:1.4	Opsoclonus, myoclonus, ataxia, cognitive and behavioural impairment associated with low-titre antibodies	Acute imaging may be normal, with later cerebellar and cortical volume loss		Neuroblastoma in about half of the children	Not known

*AMPAR* α-amino-3-hydroxy-5-methyl-4-isoxazolepropionic acid receptor, *CASPR2* contactin-associated protein 2, *CNS* central nervous system, *CSF* cerebrospinal fluid, *DPPX* dipeptidyl-peptidase-like protein-6, *D2R* dopamine 2 receptor, *F* female, *FLAIR* fluid-attenuated inversion recovery, *GABA* γ-aminobutyric acid receptor, *GAD* glutamic acid decarboxylase, *GluRD2* glutamate receptor delta 2, *GlyR* glycine receptor, *HLA* human leucocyte antigen, *LE* limbic encephalitis, *LGI1* leucine-rich glioma-inactivated 1, *M* male, *mGluR* metabotropic glutamate receptor, *MRI* magnetic resonance imaging, *NMDAR N*-methyl-d-aspartate receptor, *PERM* progressive encephalomyelitis with rigidity and myoclonus, *PNS* peripheral nervous system, *SCLC* small cell lung cancer

In clinical practice, it is well recognised that a proportion of patients present with similar features and do not have detectable autoantibodies. These ‘phenocopies’ are typically diagnosed as having a seronegative AE, and it is presumed that they have as-yet unrecognised neuronal surface autoantibodies [25, 28, 29]. This concept has been recently highlighted by a study describing 38 patients with immunotherapy-responsive brainstem–cerebellar presentations and opsoclonus, plus ovarian teratomas, yet absent NMDAR antibodies [30]. In such patients, it remains essential to make a clinical diagnosis [28], exclude alternate aetiologies, and manage these patients with immunotherapeutic treatment algorithms which are based on those with proven neuronal surface autoantibody-associated syndromes. Indeed, recent consensus criteria have been developed with ‘possible’, ‘probable’, and ‘definite’ classifications of the disease to promote instigation of immune treatments on the balance of clinical probabilities, while investigations are arranged [28].

## Signature features of NMDAR-antibody encephalitis

**Fig. 2:**
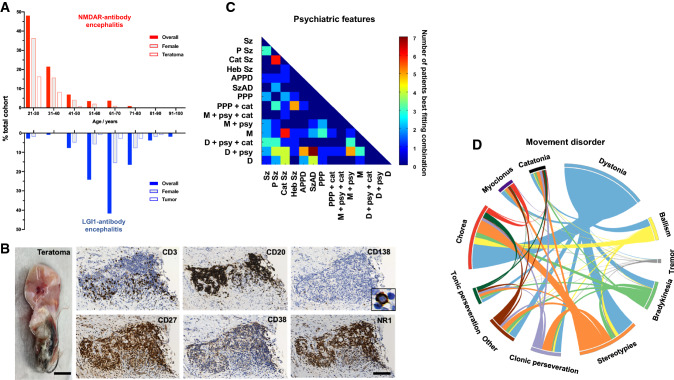
Characteristic aspects of NMDAR-antibody encephalitis. **a** Comparison of age, sex, and tumour association in adult patients with NMDAR (red) versus LGI1-antibody encephalitis (blue). **b** The ovarian teratomas associated with NMDAR-Ab-E can contain germinal centre-like structures. Here, T (CD3) and B (CD20) cell lineages are present, along with classical plasma cells (CD138), markers of T and B cell subsets (CD27 and CD38), plus the target antigen, the NR1 subunit of the NMDA Receptor. Reproduced with permission from [97]. **c** The presentation of NMDAR-Ab-E typically includes psychiatric features and movement disorders. These are both characterised by high levels of complexity, blending phenotypes which are usually discrete in individual patients. Here, a sub-group of individually reported patients (*n *= 115) with ≥ 6 psychiatric features are compared with 14 primary psychiatric disorders using constrained combination analysis. The heat map shows the pairs which best describe the data from [47]. **d** The movement disorder similarly combines multiple phenomenologies including stereotypies, dystonia and chorea. Modified with permission from [46]. *APPD* acute polymorphic psychotic disorder, *Cat Sz* catatonic schizophrenia, *D* depression, *Heb Sz* hebephrenic schizophrenia, *M* mania, *PPP* postpartum psychosis, *P Sz* paranoid schizophrenia, *Sz* schizophrenia, *SzAD* schizoaffective disorder, +* cat* with catatonia, +*psy* with psychotic features

NMDAR-antibody encephalitis is one of the most common autoimmune encephalopathies [15, 31]. While it can affect all ages and sexes, it most frequently presents in young females. It is associated with ovarian teratomas in 20–40% of cases, but has a greater female preponderance even when this is taken into account. This demographic pattern is in direct contrast to the other common AE syndrome, associated with LGI1 antibodies (Fig. 2A and Table 1). In addition to this separation, patients with LGI1 antibodies are rarely of African or Caribbean descent, whereas patients with NMDAR antibodies are often within this ethnicity [8, 16].

The clinical syndrome and the associated NR1 subunit IgG antibodies were characterised as recently as the mid-2000s [32–36]. Yet, while newly described, it is most likely not a de novo clinical disease entity. The characteristic progressive pattern of abrupt-onset bizarre behaviour, typically followed a few days later by abnormal movements, seizures, dysautonomia, and disruption of consciousness, is reminiscent of multiple syndromes previously designated as malignant catatonia, dyskinetic encephalitis lethargica, acute juvenile female non-herpetic encephalitis, and even preternatural explanations such as demonic possession [37–39]. Despite greatly increased awareness over the past decade, this symptomatic overlap with medical and non-medical concepts beyond the scope of neurology continues to risk delay in diagnosis and instigation of disease-modifying therapies.

Since the initial descriptions of NMDAR-antibody encephalitis, our understanding of the illness has been refined. This has included the identification of cases in both sexes and most commonly, without tumours; the recognition of herpes simplex encephalitis as a trigger; and the role of lymphocyte-targeted second-line immunotherapies as effective in relapse prevention [16, 40–42]. Our group and others have systematically studied the two most prominent and common clinical manifestations of this disease: the psychiatric features and movement disorders. The primary aim of such studies is not only to improve both positive identification of patients with an immunotherapy-responsive syndrome, but also to limit overconsideration of the diagnosis in presentations where the autoantibodies are not clinically relevant. In summary, the main finding has been that NMDAR-antibody encephalitis is associated with highly complex phenotypes that sample aspects from multiple sub-syndromal domains [43]. For example, the movement disorder shows almost all recognised movement disorder phenomenologies, albeit with markedly differing frequencies [44–46]. Most commonly, the movement disorder is best described by a combination of dystonia, stereotypy, and chorea with little tremor (Fig. 2b) [46]; these phenomenologies are very rarely seen together in other neurological conditions making this combination highly distinctive for NMDAR-antibody encephalitis. In an analogous manner, the mental state shows a pattern that fits poorly with primary psychiatric features and a study highlighting the psychopathological features across 464 individual patients with NMDAR-antibody encephalitis described a highly mixed mood–psychotic disorder with prominent disorganisation that only a combination of mixed primary disorders could adequately model [47]. Strikingly, an individual patient can manifest up to twenty different psychiatric features. These patterns, while complex over the time course of the disease, show strong levels of coherence indicative of consistency between patients (Fig. 2b). Recent observations also suggest that other distinctive findings in the acute phase include wandering, behavioural regression, and an abnormal sense of strength [130]. Taken together, we suggest that this stereotyped complexity can help clinicians in emergency medicine, psychiatry, and neurology develop an index of suspicion at the earliest clinical phases and, therefore, arrange for paired serum–CSF antibody testing, and prompt consideration of empirical immunotherapies [48–50]. This is critical as widespread testing of serum without refining the clinical syndrome can lead to conflation of other neurological and psychiatric disorders with NMDAR-antibody encephalitis and downstream errors of omission (lack of management of the actual diagnosis) and commission (administration of immunotherapy and exposure to side effects without a clear indication) [23, 51].

Application of contemporary molecular and systems neuroscience continues to expand our understanding of how autoantibody–receptor interactions can provoke such a clinically distinctive disease. Indeed, it is of relevance that ketamine closely mimics several aspects of the psychopathology and movement disorders associated with NMDAR-antibody encephalitis, suggesting that modification of the NMDAR alone may be sufficient to explain the clinical observations [10, 35]. Appreciating receptor-level modifications may assist in identifying optimal symptomatic therapies, as well as developing new approaches targeting the stabilisation of NMDAR-expressing synapses. For example, single-molecule microscopy techniques have demonstrated that loss of synaptic NMDAR localisation, via disruption of NMDAR–EphB2 interactions and postsynaptic density organisation, is a key pathogenic mechanism [52–54]. Importantly, these studies have demonstrated that the effect of the NMDAR autoantibodies can be partially ameliorated with pretreatment using the Ephrin-B2 receptor–ligand. This could represent an important adjunctive approach, especially when neuropsychiatric symptoms persist despite extensive immunotherapy.

Nano-level results have been contextualised by interrogation of regional connectivity plus grey and white matter integrity with MRI. The main findings include largely preserved grey matter, a degree of white matter damage, but widespread disruption of network connectivity [55, 56]. A network model of brain dysfunction provides a credible synthesis of how changes at the level of synaptic organisation can lead to wide ranging clinical manifestations in the absence of major abnormalities on routine clinical brain imaging. The role of white matter abnormalities hints at effects via glia or perhaps bystander inflammation, and indeed oligodendrocytes do express NMDARs [57]. Alongside these, the development of simple predictive clinical tools such as the anti-NMDAR Encephalitis One-year Functional Status (NEOS) Score and the Clinical Assessment Scale in Autoimmune Encephalitis (CASE), to predict outcomes and trajectories, respectively, is an important step to clinically ground a potentially molecular approach [58, 59]. Collectively, these studies set the scene for precision brain-targeted therapies, in both acute and convalescent phases. They also reinforce the need for early diagnosis and treatment to prevent disease progression during which the possibility of reversibility reduces alongside an increased likelihood of longer term cognitive burden and psychosocial dysfunction.

## Distinctive features of LGI1–antibody encephalitis

**Fig. 3:**
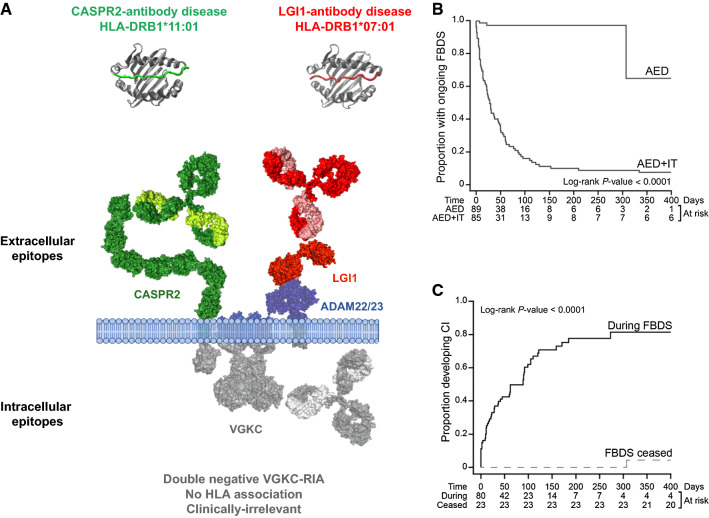
Genetic, biochemical and therapeutic aspects of LGI1, CASPR2 and double-negative voltage-gated potassium channel-complex antibodies. **a** CASPR2 and LGI1 are cell-surface-exposed proteins complexed with voltage-gated potassium channels (VGKC). LGI1 is a secreted protein which binds a disintregin and metalloprotease (ADAM) 22 and 23. The patients typically have immunotherapy-responsive diseases with strong HLA associations. In contrast, patients with VGKC-complex antibodies but without LGI1 or CASPR2 reactivities frequently bind intracellular components, do not have specific HLA associations and do not have characteristic immunotherapy-responsive clinical associations. **b** Faciobrachial dystonic seizures (FBDS) are highly responsive to immunotherapy (IT) but not to antiepileptic drugs (AED) alone. **c** Often, FBDS precede the onset of cognitive impairment. Achieving cessation of FBDS usually prevents progression to cognitive impairment. **b** and **c** Reproduced with permission from [8]. *RIA* radioimmunoassay, *AED* antiepileptic drug, *IT* immunotherapy, *FBDS* faciobrachial dystonic seizures, *CI* cognitive impairment

Antibodies targeting the voltage-gated potassium channel (VGKC) were originally described in three neurological syndromes: LE—a primarily CNS disorder; neuromyotonia—a predominantly peripheral nervous system (PNS) disorder characterised by peripheral nerve hyperexcitability, muscle cramps, and dysautonomia; and Morvan’s syndrome—an overlap syndrome with clear evidence of both CNS and PNS manifestations [60]. A radioimmunoprecipitation assay with α-dendrotoxin-labelled VGKCs extracted from mammalian brain lysates was used to detect antibodies to the VGKC. Subsequently, it was discovered that, in fact, these antibodies bound to proteins which were often complexed with the VGKCs: LGI1, contactin-associated protein 2 (CASPR2) and, more rarely, contactin-2 [60, 61] (Fig. 3a). VGKC antibodies which do not bind to one of these proteins (often termed ‘double negative’) are far more common than LGI1 or CASPR2-reactivities [62, 63], are not associated with a defined immunotherapy-responsive clinical syndrome, have low syndrome specificity, and appear to target cytosolic epitopes unrelated to disease pathogenesis [60, 64–66]. In addition to this issue around specificity of VGKC–antibodies, the VGKC assay fails to detect a number of LGI and CASPR2-specific autoantibodies, meaning that it also has reduced sensitivity. Taken together, these findings indicate that VGKC–antibody testing is clinically obsolete and has no place in routine diagnostic laboratories. Rather, patients with a suggestive clinical presentation should be specifically tested for LGI1 or CASPR2 antibodies.

LGI1-antibody encephalitis is most common in the elderly with a significant male preponderance [63, 67]. There is an annual incidence of approximately 1–2 per million per year (Irani and Waters, unpublished observations) [67]. Genetic studies have shown that around 90% of patients with LGI1 antibodies from both Caucasian and Asian cohorts [68–70] carry the human leukocyte antigen (HLA) DRB*07:01.

The two cardinal clinical features of LGI1-antibody encephalitis are seizures and cognitive impairment [60, 61, 67]. The pathognomonic seizure syndrome for this condition is termed faciobrachial dystonic seizures (FBDS) and has only been recognised in association with LGI1 antibodies to date. This specificity may be akin to the stereotyped complexity of movement disorders and psychopathology closely associated with NMDAR-antibody encephalitis. FBDS refer to brief stereotyped events, most commonly involving the hemiface and ipsilateral arm which occur at a median of 50 times per day at disease nadir [71, 72]. They are associated with classically epileptic ictal features such as speech arrest, agitation, fear, and automatisms [71, 72]. FBDS often precede the development of the cognitive impairment seen in patients with LGI1 antibodies which predominantly involves episodic memory and executive function [27, 72–74]. Other frequent focal seizure semiologies which may occur concurrently with, or in the absence of FBDS, include thermal sensations, body shuddering, motor automatisms, gelastic seizures, and paroxysmal dizzy spells [27, 62, 75–77]. Generalised tonic–clonic seizures are uncommon and usually manifest in the later stages of disease. Importantly, focal seizures—in particular FBDS—are often the first feature of this illness. Hence, their early clinical recognition is paramount prior to the development of a fulminant encephalitic syndrome.

Ancillary investigations may be non-contributory, with EEG often being non-specific and over 50% of patients having normal MRI and CSF analysis [8, 27, 72, 75]. This highlights the importance of the syndrome being a predominantly clinical diagnosis confirmed by detection of LGI1-specific antibodies in the serum, and at a lower frequency in CSF [27]. Where present, ancillary findings may include serum hyponatraemia and MRI evidence of medial temporal swelling during the acute presentation, with longer term development of mesial temporal sclerosis [27, 60, 61, 75]. Exclusively in patients with FBDS, basal ganglia signal changes contralateral to the side of clinical involvement are well described [62, 72, 73, 78, 79]. LGI1 antibodies only rarely associate with tumours, usually thymomas, which are more common in the patients with both LGI1 and CASPR2 antibodies [62].

LGI1-antibody encephalitis is most convincingly demonstrated to be an immunotherapy-responsive clinical syndrome by a large cohort of patients with FBDS who had refractory seizures when treated with (often several) antiepileptic drugs alone [8]. By contrast, 90% had a favourable response to the initiation of immunotherapy, which was often very rapid, sometimes occurring a few days after commencement of corticosteroids [8, 62] (Fig. 3b). As FBDS and other focal seizure syndromes commonly precede the onset of cognitive symptoms, their clinical recognition provides a critical therapeutic window to expedite diagnosis and initiation of immunotherapy, with cessation of FBDS shown to prevent the long-term functionally impairing sequelae of cognitive impairment (Fig. 3c) [8]. In addition, rapid corticosteroid withdrawal often appears to predispose to early relapses [27, 72]. By contrast, our clinical observation is that patients who receive longer term immunotherapies, have very few relapses. However, as only small numbers of reported patients have been administered chronic immunosuppression with steroid sparing agents, such as rituximab and cyclophosphamide, the therapeutic efficacy of these agents is currently difficult to more formally determine [80, 81].

LGI1- and CASPR2-antibody encephalitis syndromes share significant overlap with regard to clinical and radiological phenotypes (Table 2), yet patients with CASPR2 antibodies tend towards the neuromyotonia and Morvan’s phenotypes, and often have underlying thymomas [81, 82]. The more precise phenotypic differences await formal description although neuropathic pain syndromes appear to be more common in patients with CASPR2 antibodies, and, to date, these patients have not been reported to have FBDS without concomitant LGI1 antibodies.

## Differential diagnoses

While viral encephalitis may be considered in the differential diagnosis of a patient with an acute-onset encephalopathy, its presentation differs from AE with a higher likelihood of fevers, CSF inflammation, and the absence of the signature neuropsychiatric, seizure, and movement disorder manifestations seen in patients with LGI1- or NMDAR-antibody encephalitis [7]. Differential diagnoses apart from established neuronal surface antibody syndromes and seronegative AE include rare CNS inflammatory disorders such as Hashimoto’s encephalopathy, Rasmussen’s encephalitis, Bickerstaff’s encephalitis, and progressive encephalomyelitis with rigidity and myoclonus [28], as well as prion disease such as Creutzfeldt–Jakob disease [83]. Drugs which result in pharmacological disruption of the NMDAR, such as ketamine, may present similar to NMDAR-antibody encephalitis [35]. Recent expert consensus suggests that AE should be considered in patients with subacute memory impairment and psychiatric symptoms, plus at least one of the following: new focal CNS findings, seizures without an alternate aetiology, MRI consistent with encephalitis, and exclusion of alternative diagnoses [28].

## Therapeutic paradigms in autoimmune encephalitis

Multicentre observational studies involving relatively large cohorts of patients with NMDAR- and LGI1-antibody encephalitis show intrinsic methodological biases, but highlight common themes in therapeutic efficacy and outcomes, which we can reasonably extrapolate to the treatment of other forms of AE [3, 84].

**Fig. 4:**
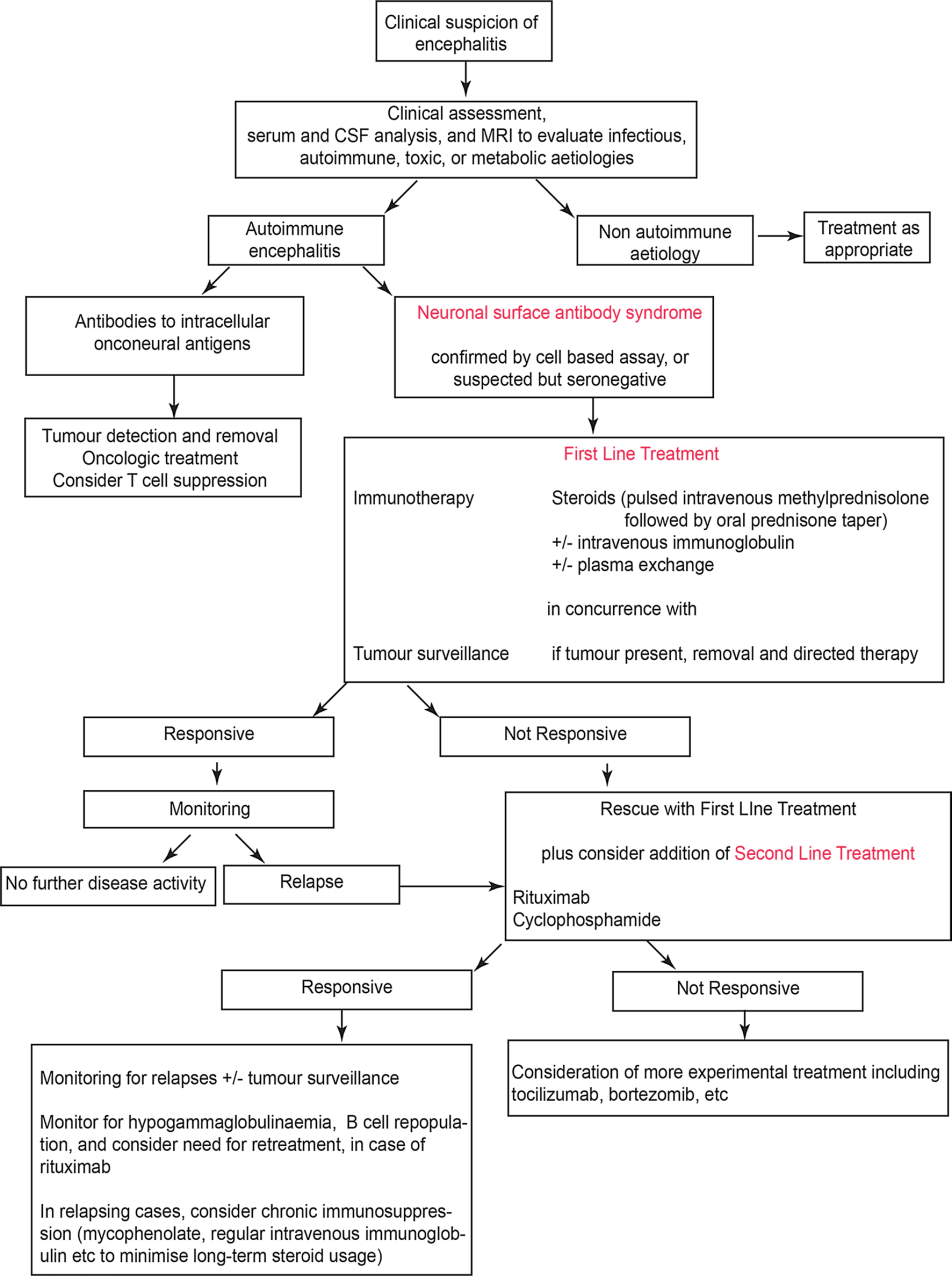
A diagnostic and therapeutic approach to suspected autoimmune encephalitis Adapted with permission from [7]

First-line therapy is often in the form of pulsed intravenous methylprednisolone (often followed by high-dose oral prednisone), plus plasmapheresis and/or intravenous immunoglobulin [7, 16]. In addition, early tumour surveillance is recommended as tumour-directed therapy can be important [6, 16, 40]. Failure to respond to first-line agents should lead to therapy escalation including further steroids/plasmapheresis plus consideration of second-line therapies including rituximab or cyclophosphamide [4, 29, 84, 85]. The interval between waiting for first-line therapy to take effect and commencing second-line therapy is debated, and may be reasonably dictated by the severity of presentation, the degree and rate of improvement, and the relative clinical experience of different centres. However, a critical theme in the treatment of AE is that the early institution of immunotherapy is closely linked to reducing long-term sequelae and relapses, and improving outcomes in the short and longer terms [8, 12, 16, 80, 84]. A minority of patients remain refractory to second-line therapy and in a small number of these patients, case reports and case series have highlighted the possible use of third-line immunotherapies such as tocilizumab and bortezomib [86, 87]. These principles are illustrated in a proposed therapeutic algorithm (Fig. 4) but currently lack a robust immunomechanism-based set of concepts for the choice of immunotherapy.

## Proposed pathophysiological mechanisms of action of autoantibodies

**Fig. 5:**
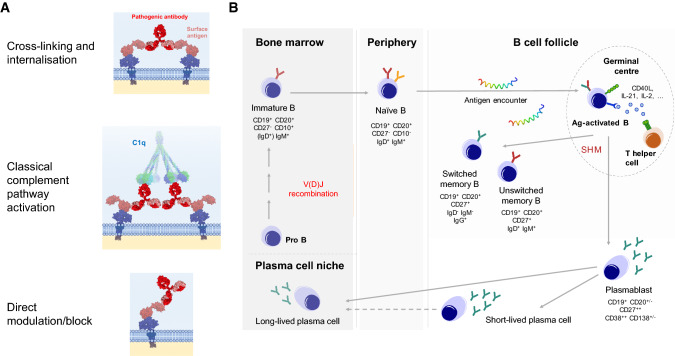
Potential pathogenic mechanisms of neuronal surface antibodies and B cell lineages underlying generation of antibody-secreting cells. **a** Antibodies against neuronal surface epitopes can mediate pathogenic effects through multiple mechanisms which include cross-linking and internalisation of the target, fixation of C1q and activation of the classical complement pathway, and direct interference with channel function including pharmacological-type block. **b** B cells are formed from haematopoietic stem cells in the bone marrow and undergo recombination of V, D and J immunoglobulin genes to generate a functional B cell receptor. They enter peripheral blood becoming a naïve B cell, and in lymphatic tissue encounter cognate antigen, leading to B cell activation and generation of germinal centres. In germinal centres, B cells process antigen and present it as peptide on surface MHC molecules to T-helper cells, which in turn provide support for the activated B cells. During the process of affinity maturation, B cells undergo somatic hypermutation leading to a diversity of antigen-specific B cell receptors. Alongside this, the immunoglobulin class often switches from IgM to IgG. This reaction generates memory B cells, as well as antibody-secreting cells in the periphery (plasmablasts). Antibody-secreting cells vary in their longevity and migration back to the bone marrow to a survival niche and are associated with long-term antibody secretion. Reproduced with permission from [102]

Several in vitro and in vivo studies demonstrate potential pathogenic mechanisms of action by which neuronal surface antibodies may manifest their deleterious downstream effects. Broadly, the predominant mechanisms are target internalisation and complement activation (Fig. 5).

NMDAR IgG autoantibodies have been shown to exert a highly selective and concentration-dependent reduction of postsynaptic clusters of NMDAR in hippocampal neurons which has been observed in vitro and in vivo [88]. This is likely due to the antibody-induced internalisation of surface-expressed NMDARs [5, 34, 89, 90]. Removal of the antibodies restores the surface NMDARs, suggesting that this process is reversible, consistent with the observed improvements of patients after immunotherapy, and the absence of neuronal loss in imaging and histopathology studies. Similar phenomena of receptor internalisation have been noted with AMPAR, glycine receptor, and GABA_A_R antibodies [91–93], but not GABA_B_R antibodies [94]. In addition, using the soluble LGI1 protein and its known receptor ADAM22, we recently showed that internalisation of the LGI1–ADAM22 complex could be mediated by LGI1 antibodies [8, 91].

Finally, complement deposition may be a pathogenic mechanism in select cases. By contrast to the IgG4-dominant antibodies which target LGI1, CASPR2, and IgLON5, other pathogenic neuroglial surface receptor antibodies are of the IgG1 subclass. IgG1 antibodies have the ability to activate complement [40]. This is of interest because despite the majority of LGI1 antibodies being of the IgG4 subclass, a proportion of patients with LGI1 IgG1 antibodies are over-represented in those with cognitive impairment, suggesting that some irreversible sequelae in LGI1-antibody encephalitis may be associated with complement-mediated effects [8]. These questions are not likely to be directly addressed in future post-mortem studies, given the pleasingly low contemporary mortality associated with these diseases. Hence, in vitro studies and in vivo imaging of humans may be more informative opportunities to more formally model these observations.

## How is autoimmunity initiated in these patients?

The studies outlined above address possible mechanisms by which pathogenic autoantibodies exert their functional effects. However, they do not identify the original initiating autoimmune process. Below, we will explore identified triggers including tumours and infections, underlying genetic susceptibilities, and what is known about the production of pathogenic antibodies across stages of B cell development. These immunological observations aim to inform mechanisms underlying the propagation and the cause of these diseases.

Paraneoplastic associations are present in several neuronal surface antibody syndromes. In particular, the frequent relationship between NMDAR-antibody encephalitis and teratomas generates a compelling line of investigation. Histopathological studies of patient teratomas have highlighted dense B and T cell infiltrates, neuroglial NR1 antigen expression plus dysplastic neuronal elements (Fig. 2c) [95–97]. These observations imply ectopic antigen expression and reactive germinal centre reactions in NMDAR-antibody encephalitis–teratomas, which may be important for disease induction [97]. Furthermore, and more definitively pathogenic, teratoma explants can produce the NMDAR antibodies in vitro, suggesting intratumoural B cells have the capacity to secrete pathogenic antibodies [97]. Taken together with the time-dependent clinical efficacy of oophorectomy in improving outcomes [16, 34, 40], this suggests that the initiation of the immune process occurs in the peripheral compartments with intratumoural synthesis, and the subsequent passage of B cells to the circulation, and the intrathecal space. Indeed, this model aligns well with the consistently higher serum levels of autoantibodies in these conditions [24, 25, 27, 40].

In support of this peripheral-to-central flux of the immune process, a recent clinical observation has shed light on the exposure of brain antigens to the immune system as a potential trigger of AE. Patients with proven herpes simplex virus encephalitis (HSVE) have been noted to develop a clinical worsening a few weeks after initiation of their HSVE. This worsening is associated with the de novo generation of autoantibodies [41, 42, 98, 99]. Around 50% of patients seroconvert, with generation of several surface neuronal autoantibodies during the first few months of HSVE [25]. Around 50% of the autoantibodies had NMDAR reactivity, but other non-NMDAR targets have also been found, including D2R, AMPAR, and GABA_A_ receptor antibodies plus surface neuronal binding without an identified target [3, 25, 42, 100]. HSVE animal models have mimicked this seroconversion with the generation of serum NMDAR antibodies after intranasal HSV inoculation [101]. There have also been reports of NMDAR antibodies following other CNS viral infections, which argue against classical molecular mimicry being a dominant mechanism in this natural human immunisation paradigm [100]. Indeed, generic viral nucleic acid mimics are known to efficiently stimulate circulating B cells to produce antigen-specific autoantibodies [97, 102], adding to the evidence that viral infections may contribute directly to autoimmunity. A parsimonious explanation is that neuronal damage, the inflammatory milieu, and the virus directly may expose previously sequestered brain antigens to reactive lymphocytes within draining cervical lymph nodes, thereby overcoming the status quo of CNS immune privilege and generating brain-specific autoantibodies. Yet, how the antigen-specific lymphocytes originally escape immunological tolerance has been studied in only a few patients to date.

We have identified naïve pre-germinal centre B cells capable of differentiating to secrete aquaporin-4-specific autoantibodies in patients with another CNS autoantibody-mediated condition, neuromyelitis optica [102]. Therefore, preformed B cells can possess antigen-specific reactivity. This may also be the case in NMDAR-antibody encephalitis where some monoclonal NR1-specific antibodies derived from patient CSF B cells showed no or few somatic hypermutations [26]; these antibodies were able to bind NMDARs and mediate functional effects in vitro [103]. This also suggests that a naïve repertoire of antigen-specific B cells is present in patients. However, by contrast to aquaporin-4 antibodies, this initial report suggests that the NMDAR antibodies do not appear to mutate heavily, even once in the intrathecal space. Yet, the teratoma histology provides strong evidence for a germinal centre, a classical site of hypermutation. Circulating NR1-IgG concentrations are proportional to the ex vivo capacity of patient B cells to secrete NR1 IgGs, again suggesting cells derived from germinal centre spillover account for serum NR1 IgGs [97]. Therefore, the relative roles of naïve B cells and hypermutated memory B cells remain to be established and may have implications for optimal tailored therapies or identification of ‘at risk’ patients.

Studies in patients with LGI1 and CASPR2 antibodies have also implied a role for germinal centre reactions, via the identification of strong and dichotomous genetic susceptibilities. Over 90% of LGI1-antibody-positive encephalitis patients, of both East Asian and Caucasian extraction, have the HLA-DRB1*07:01allele [68–70], while patients with CASPR2-antibody encephalitis have a marked overrepresentation of HLA DRB1*11:0l [70]. There is a strong correlation between IgLON5 antibodies and HLA-DRB1*10:01-DQB1*05:01 [104], and a weak association with NMDAR-antibody encephalitis and the HLA-B*07:02 allele [105]. While none of the alleles are likely to be the sole susceptibility factor involved, these associations imply restricted T–B cell interactions are critical in the generation of autoantibodies. In addition, we are finding that these HLA associations are absent in patients with autoantibodies but without clinically compatible syndromes [70], suggesting that they may emerge as useful ancillary tools in clinical diagnosis.

## Conclusion

Many of the early clinical observations in AE have been further refined to permit the accurate recognition of distinctive clinical phenotypes. This serves as a starting point not only to guide clinical practice, but also to inform focussed laboratory lines of enquiry. The relative rarity of these conditions on a population level remains a challenge to research, and highlights the notion that international and multicentre collaborations remain essential to move the field forward. AE is now identified as an important and previously under-recognised cause of CNS inflammation—it is highly treatable, and early diagnosis and initiation of immunotherapy are paramount to optimise outcomes. The field is now entering a more mature phase of research with advanced approaches in imaging, molecular immunology, and neuroscience. This will ideally permit a more cohesive understanding of aetiology and pathogenesis, and ultimately advance precision therapy in the future.
